# Development of wheelchair caster testing equipment and preliminary testing of caster models

**DOI:** 10.4102/ajod.v6i0.358

**Published:** 2017-09-28

**Authors:** Anand Mhatre, Joseph Ott, Jonathan Pearlman

**Affiliations:** 1 Human Engineering Research Laboratories, Department of Veterans Affairs, Pittsburgh, United States; 2 Department of Rehabilitation Science and Technology, University of Pittsburgh, United States; 3 International Society of Wheelchair Professionals, University of Pittsburgh, United States

## Abstract

**Background:**

Because of the adverse environmental conditions present in less-resourced environments (LREs), the World Health Organization (WHO) has recommended that specialised wheelchair test methods may need to be developed to support product quality standards in these environments. A group of experts identified caster test methods as a high priority because of their common failure in LREs, and the insufficiency of existing test methods described in the International Organization for Standardization (ISO) Wheelchair Testing Standards (ISO 7176).

**Objectives:**

To develop and demonstrate the feasibility of a caster system test method.

**Method:**

Background literature and expert opinions were collected to identify existing caster test methods, caster failures common in LREs and environmental conditions present in LREs. Several conceptual designs for the caster testing method were developed, and through an iterative process using expert feedback, a final concept and a design were developed and a prototype was fabricated. Feasibility tests were conducted by testing a series of caster systems from wheelchairs used in LREs, and failure modes were recorded and compared to anecdotal reports about field failures.

**Results:**

The new caster testing system was developed and it provides the flexibility to expose caster systems to typical conditions in LREs. Caster failures such as stem bolt fractures, fork fractures, bearing failures and tire cracking occurred during testing trials and are consistent with field failures.

**Conclusion:**

The new caster test system has the capability to incorporate necessary test factors that degrade caster quality in LREs. Future work includes developing and validating a testing protocol that results in failure modes common during wheelchair use in LRE.

## Introduction

There is an estimated unmet need of 95 million wheelchairs worldwide (Borg & Khasnabis 2008; Handicap International 2013; Mhatre et al. 2017; World Health Organization 2011). To address this need in less-resourced environments (LREs), various international organisations such as the World Health Organization (WHO), United Nations (UN), United States Agency for International Development (USAID) and International Society of Wheelchair Professionals (ISWP) are promoting increased access to appropriate wheelchairs (Borg & Khasnabis 2008; International Society of Wheelchair Professionals 2015; United Nations 2006; United States Agency for International Development 2016). The UN launched the Convention for Rights of People with Disabilities (CRPD) in 2006, and Article 20 of the CRPD highlights the need for access to appropriate and high-quality mobility aids that include wheelchairs (United Nations 2006). During the same year, a consensus conference was held that brought together groups involved in the provision of wheelchair services in LREs and wheelchair experts to discuss wheelchair needs and issues with products and services (Sheldon & Jacobs 2006). The outcomes of this conference influenced WHO in developing the Guidelines on Provision of Manual Wheelchairs in Less-resourced Settings (WHO Guidelines) and the wheelchair service training packages in partnership with the USAID (Borg & Khasnabis 2008; World Health Organization 2012, 2013). Recently, the WHO in collaboration with the UN and USAID initiated the Global Cooperation on Assistive Technology (GATE) programme to accelerate CRPD initiatives (World Health Organization 2014) and published a Priority Assistive Products List that emphasises the access for high-quality products and recognises four distinct wheelchair designs as appropriate products for provision. Provision of appropriate wheelchair designs has been advocated for by the WHO Guidelines too. Furthermore, recommendations by the WHO have guided the development of wheelchair-related initiatives of ISWP. ISWP aims at benefitting both wheelchair users and providers by promoting wheelchair provision training and research activities, improving wheelchair design and manufacturing and coordinating wheelchair sector services. ISWP initiatives are guided by an advisory board consisting of international wheelchair experts, and there are several working groups that develop and implement action plans for improving services and products. ISWP is currently developing and conducting wheelchair provision knowledge and skills tests internationally, developing tools and resources for wheelchair users and providers, collaborating with various organisations internationally and creating awareness in the wheelchair community to enhance the overall quality of wheelchair services and thereby addressing the unmet need for wheelchairs in LREs (International Society of Wheelchair Professionals 2015).

**FIGURE 1:**
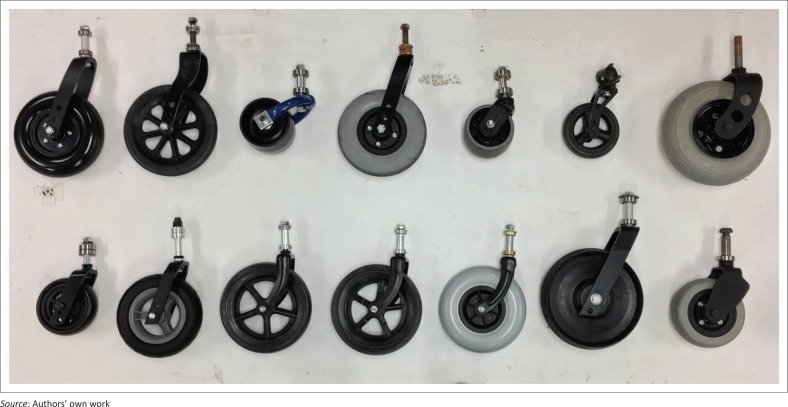
Different caster designs used on wheelchairs.

**FIGURE 2:**
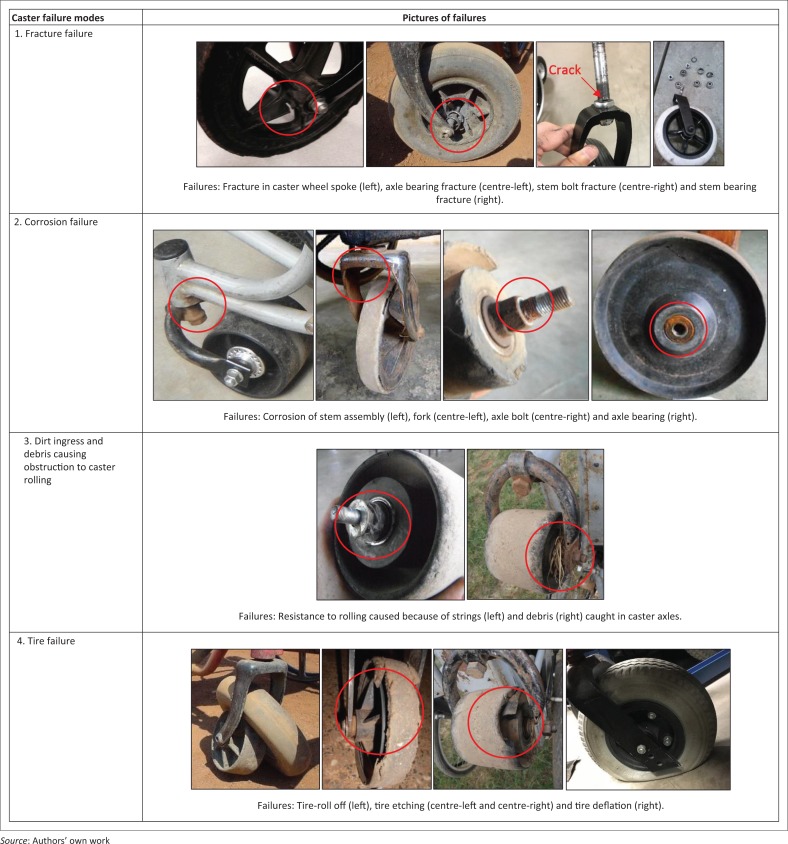
Caster assembly failures witnessed in the field.

There are several challenging issues in LREs related to wheelchair policies, services, products and awareness, and one of the greatest concerns is quality of products. Most LREs fall in the tropical zone (Monk & Wee 2008) and experience high temperature and humidity, which adversely affects product quality. Moreover, outdoor terrains and wheelchair use conditions evident in LREs impose greater quality requirements on products, which are known causes for wheelchairs to fail prematurely (Constantine, Hingley & Howitt 2006; Hotchkiss 1985; Mukherjee & Samanta 2005; Saha et al. 1990; Sheldon & Jacobs 2006; Shore 2008; Shore & Juillerat 2012). Wheelchair casters specifically are a common point of failures as wheelchairs travel over rocky terrains, debris and face inclement weather. A variety of caster designs are used on wheelchairs (see Figure 1), and the demanding operating conditions in LREs cause these casters to fail in different modes as shown in Figure 2.

**FIGURE 3:**
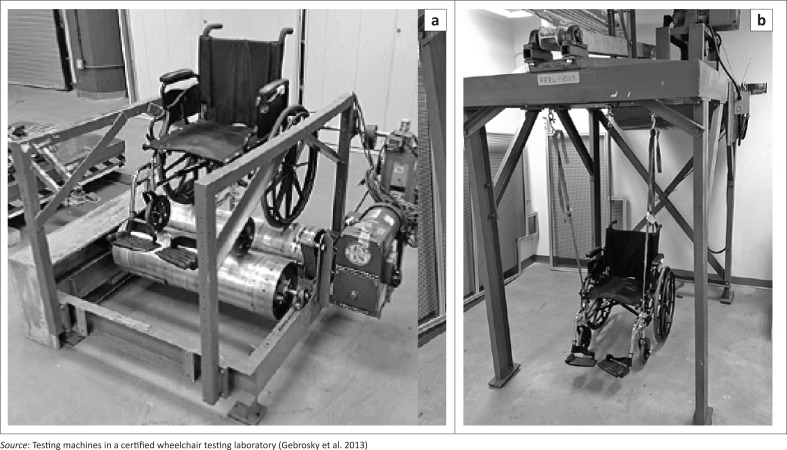
Multi-drum test (left) and curb-drop test (right) without test dummies.

Caster quality issues have been found during standardised wheelchair tests published by the International Organization for Standardization (ISO). ISO 7176 standard Section 8 refers to durability testing of wheelchairs that includes static, impact and fatigue tests (International Organization for Standardization 2014). Fatigue tests subject wheelchairs to 200 000 drum revolutions (or test cycles) during multi-drum tests (MDT) and 6667 drops during curb-drop tests (CDT) (see Figure 3). Casters are known to undergo fracture failures throughout such tests. Fractures with caster’s vertical stem assemblies are common (Cooper et al. 1996, 1997; Cooper, Boninger & Rentschler 1999; Cooper, Stewart & VanSickle 1994; Fitzgerald et al. 2001; Kwarciak et al. 2005). Fractures with the caster fork, bearings and wheel spokes have been reported too, as have alignment issues with the caster wheel (Cooper et al. 1994, 1996; Toro et al. 2013). Additionally, failures and repairs with casters have been noted in field studies that evaluated usability and performance of wheelchair products (Armstrong, Reisinger & Smith 2007; Mukherjee & Samanta 2005; Reese & Rispin 2015; Saha et al. 1990; Shore & Juillerat 2012; Toro et al. 2012). In one study, casters were found to be a constant source of worry; Saha et al. (1990) reported breaking caster wheels and forks, missing tires and bolts, locking of the casters while rolling, bearing failures and excessive caster vibration with LRE- produced chairs. Casters sinking into soft ground and failure while climbing over obstacles were some of the performance issues caused by inappropriate product design. Premature wheelchair breakdown occurred, as casters were found to not last more than 6 months (Saha et al. 1990). In another study, caster tires were found to crack and wear within 1–2 years of use (Reese & Rispin 2015). Most caster failures are known to cause user discomfort, adverse incidences such as accidents leading to user injuries and wheelchair breakdowns (Gaal et al. 1997; Saha et al. 1990). In the LRE context, broken parts are often difficult to repair or replace. Lack of resources (rehabilitation services and skilled labour), availability of replacement parts, user training during provision and awareness create challenges for user maintenance, servicing and repair (Borg, Lindström & Larsson 2011; Pearlman et al. 2008). Product breakdowns can cause long-term loss of mobility, because users in LREs often do not have a back-up wheelchair (Hotchkiss 1985). This in turn has multi-dimensional consequences for the user, including reduced satisfaction and likelihood of wheelchair abandonment (Fitzgerald et al. 2005; Phillips & Zhao 1993).

To facilitate the necessary provision of high-quality wheelchairs and casters, the WHO Guidelines suggest products undergo ISO 7176 standard wheelchair testing prior to their sale and distribution. Furthermore, to address the diverse and demanding LRE conditions, the WHO Guidelines recommend the development of additional tests (Borg & Khasnabis 2008). While the purpose of such testing is well understood, there are no clear directions on the design and development of these tests. The development work for additional tests was taken up by ISWP’s Standards Working Group (ISWP-SWG), which includes wheelchair experts that have considerable industry and fieldwork experiences in LREs (Mhatre et al. 2017). This group was formed to enhance product quality, as well as to develop standards and resources to promote appropriate high-quality products for delivery in LREs. Among the additional tests that the group has proposed, caster durability testing was ranked (through consensus voting) as one the most critical areas for testing (Mhatre et al. 2017). This article covers the design process followed by the ISWP-SWG for developing new caster testing equipment and the preliminary testing that was conducted with different caster designs.

## Methods

The development of additional tests commenced in early 2015 after the ISWP-SWG discussed the concept. The group members reported several wheelchair parts failures evident in LREs, identified factors that contribute to field failures and evaluated whether these factors are included in ISO 7176 fatigue tests of MDT and CDT. The results of this evaluation for casters demonstrated the lack of requisite test factors in standard testing of caster assemblies which implied developing a new testing method.

For developing additional tests, the ISWP-SWG was divided into subgroups and the caster testing subcommittee led the development of new caster testing method. Searches were conducted for standards available for caster assembly testing. The results were obtained and reviewed by the authors for relevant testing methods. Other testing methods for wheelchair casters were retrieved from literature review work conducted by the authors previously (Mhatre et al. 2017). ISWP-SWG members reported on caster testing systems developed by wheelchair manufacturers. Testing methods retrieved from different sources were evaluated for presence of testing factors pertaining to LRE conditions. The result of this search process informed the group that a new testing system needs to be developed.

The caster test design process began with ISWP-SWG experts putting together the functional requirements for the new testing method. The requirements were based on the gaps in current caster testing methods (ISO and other standards) and the expected testing conditions corresponding to LRE use. The members of the caster testing subcommittee developed design concepts accordingly. Feedback on the designs was taken in three steps. Firstly, through ISWP-SWG discussions, the advantages and disadvantages of each concept were discussed in detail and a single design for further development was selected. Secondly, the designs were drafted in detail and a second round of feedback was conducted through an in-person meeting with all ISWP-SWG experts. Design recommendations were provided. Finally, the design was refined according to the recommendations, benchmarked to MDT test conditions, and further feedback was sought from the machine shop staff at the Human Engineering Research Laboratories (HERL, Pittsburgh, USA) where the final design was to be fabricated. Following approval from different contingents, the equipment was fabricated at HERL over a period of 2 months.

To evaluate testing feasibility and efficiency of the new equipment, models of casters differing in sizes and parts’ designs were tested initially. Four caster models were tested for defined number of test cycles under known weight. As impacts in the field are at different angles, casters were subjected to oblique slat impacts, except one model which was tested for square slat impact (with slats fixed at zero-degree angle). Following reliable performance of the new equipment, a preliminary testing study was carried out with six caster models to evaluate effect of straight versus oblique slat impacts on durability of caster assemblies. Four samples of each model were tested. For each model, two samples were subjected to square impacts (zero degrees) and the other two to oblique impacts at 30 degrees (±1.5% error). Casters were tested under known weight until failure in this study. A paired samples *t*-test was used to compare test cycles completed with the two slat angle conditions for each model. Caster assembly failures were documented and analysed. Feedback was sought from respective caster manufacturers about the failures seen on the caster test. Results of this testing informed the caster testing subcommittee of necessary modifications to the caster testing protocol.

## Results

### Field failures of caster assemblies

Outdoor conditions leading to field failures of casters were identified by ISWP-SWG experts. Comparing different test factors corresponding to each outdoor condition with the testing conditions on ISO 7176 fatigue tests of MDT and CDT yielded results as seen in Table 1. Several test factors of interest were not included in standards testing.

**TABLE 1:** Caster assembly failure modes and corresponding quality-affecting factors as seen in the field.

Failure modes	Outdoor factors	Factor inclusion status in ISO 7176
Broken and bent caster parts	Impacts and loads, fracture loads, oblique impacts.	Yes (ISO 7176 – 8), but MDT and CDT do not reproduce complex load conditions that occur in LREs.
Corrosion in bearings and on metallic parts	Corrosion because of high humidity environments.	Not in ISO 7176
Worn out tires	Abrasion because of rougher terrains.	Not in ISO 7176
Tire puncture	1. Rocky surfaces.	Not in ISO 7176
	2. Poor air retention capability of the tube in tire.	
Parts degradation	Accelerated aging because of ultraviolet light (UV), high temperatures and rough surfaces.	Not in ISO 7176
Fluttering caster	Caster flutter on rocky surfaces at high speed.	Seen on MDT but not tested for.
Worn out bearings	1. Poor lubrication, seal design & quality.	Not in ISO 7176
Dirt and dust in bearings	2. Heavy impacts.	
High rolling resistance	Design of caster parts not applicable to LREs.	Not in ISO 7176
Caster caught in obstacles	Design of caster parts not applicable to LREs.	Not in ISO 7176

*Source*: Authors’ own work

MDT, multi-drum tests; CDT, curb-drop tests; LREs, less-resourced environments; ISO, International Organization for Standardization; UV, ultraviolet.

### Review of caster standards, testing literature and existing test methods

**FIGURE 4:**
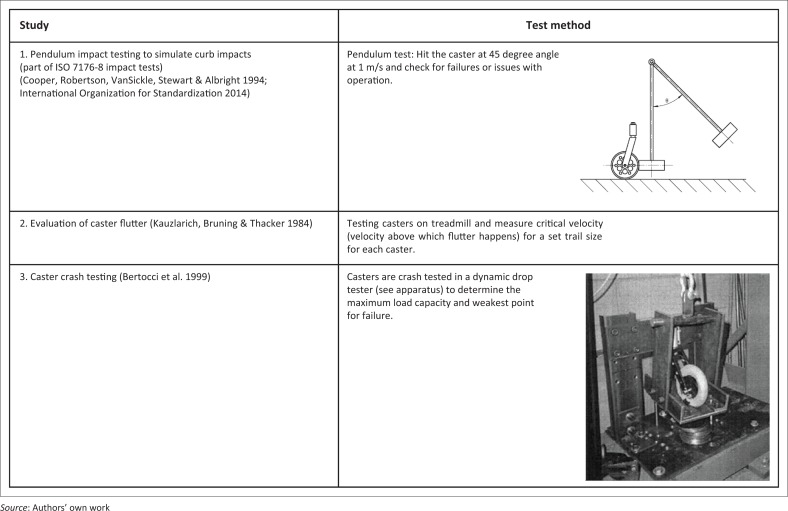
Caster test methods reported in the literature.

Caster testing standards have been published by the ISO and the American National Standards Institute – Institute of Caster and Wheel Manufacturers (ANSI-ICWM) (Institute of Caster and Wheel Manufacturers 2012). ISO 22877-82 covers standards for casters for institutional use such as furniture and swivel chairs for use in shops, restaurants, hotels, educational buildings and hospitals (International Organization for Standardization 2004b). ISO 22883-84 is suitable for casters used in industrial environments (International Organization for Standardization 2004a). The ISO standards contain methods for fatigue and performance testing of caster braking systems, but testing methods for durability testing of the entire caster assembly have not been included. The ANSI-ICWM standards contain static load tests, side load tests and vertical impact tests for industrial and institutional casters (Institute of Caster and Wheel Manufacturers 2012). Dynamic tests are included, and they qualify as durability tests. They require casters to roll over obstacles (obstacle height based on caster diameter) and multiple track configurations that include a linear track, circular track (horizontal position) and circular track (vertical position). Testing methods that were found in the literature are listed in Figure 4.

**FIGURE 5:**
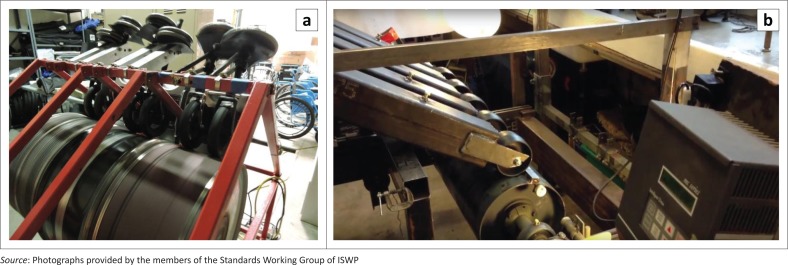
Caster testing drum equipment with wheelchair manufacturers.

The development of caster testing machines by two wheelchair manufacturers was reported by ISWP-SWG members. These included weighted casters mounted on a drum with a slat (similar to MDT test) (Figure 5). However, caster testing methods with appropriate test factors relevant to field use in LREs were not found in the standards, literature or any searches.

### Development of new caster test system

Following review of existing caster testing methods, the ISWP-SWG decided on developing new caster assembly testing equipment which could incorporate relevant testing factors. Outcomes from comparison in Table 1 assisted in developing the functional requirements of the new system. They are as follows:

- The new testing system subjects casters to straight and oblique impacts.
- The new testing system exposes casters to a variety of surface patterns to simulate LRE terrains. Replacing a surface during testing should be easy and require minimal time and effort.
- The new testing system exposes casters to moisture/water to simulate corrosion failures.
- The new testing system tests casters of different designs at the same time for comparison testing.
- To simulate appropriate caster behaviour, the new testing mounts caster for testing similar to the way it is mounted on its wheelchair.
- The new testing system is flexible to change speed and direction during testing. The optimal speed recommended for this test was 1 m/s (same as MDT).
- The new testing system allows a range of weights for loading on casters.
- The new testing system includes an accelerated wear test for casters.
- The new testing system replicates failures as seen in the field.

The caster testing subcommittee members developed design concepts based on functional requirements. Six concepts were proposed (see Table 2) and modelled for initial evaluation by the ISWP-SWG. Advantages and disadvantages of each were discussed for selecting an appropriate concept for design and development.

**TABLE 2:** Design concepts evaluated by the ISWP-SWG experts.

Concept description	Advantage	Disadvantage	Selected for development
1. Weighted caster(s) mounted on a treadmill with bumps and rough surfaces.	Reliable system for exposure to different load conditions.	Durability concerns with the treadmill belt; it is difficult to retain rough surfaces and bumps on a rotating belt over time. Like MDT, stem bearings may not be tested as casters do not swivel about stem axis.	No
2. Weighted caster(s) tested on a reciprocating table with bumps and rough surfaces.	Change in direction is useful for testing the stem bearing assembly of the caster.	Testing multiple casters with reciprocating movement (at a speed of 1 m/s) would require a larger surface area.	No
3. Weighted caster(s) rolling on a heated drum (like MDT) with rough surfaces and slats (bumps). The caster is exposed to acidic, salt spray and UV light while running on drum.	Concept to incorporate different test factors at same time. Reliable system for testing.	Attaching different surfaces to drum’s surface is difficult. Replacing surfaces quickly during testing can be difficult and will require more time. Heating the drum is a mechanism. Salt spray and UV exposure affects strength of the test equipment.	No
4. Weighted caster(s) turning in a circle like a carousel over different rough surfaces and bumps on a table. The casters are mounted on arms that are attached to the centre shaft (see Figure 6).	Different surfaces and loads can be switched during testing. Speed and direction of the shaft changes.	Heavy weights on rotating casters at 1 m/s may be unsafe. The behaviour of a revolving caster after hitting a bump depends on speed, moment of inertia around the stem axis and load. The caster can swing out abruptly after impacts, which may not be representative of outdoor behaviour of casters.	No
5. Concept #5 is similar to #4; in this concept, the casters are stationary and the table rotates (see Figure 6).	Advantages are similar to concept #4. As casters do not revolve, they may swing out moderately.	Exposure to several test factors like humidity, UV and high temperature may be difficult with this setup as it can possibly degrade the equipment.	Yes
6. Concept #6 is similar to #4 above but the entire assembly is enclosed in a drum at an angle and partially filled with water.	Advantages are similar to concept #4. Consistent exposure to moisture.	Disadvantages are similar to that of concept #4. Weight on top of the caster will not be same at different points of travel and the caster may swing inward/outward (based on position) because of gravity after hitting bumps. Casters can remain wet throughout the test, which is not typical outdoors.	No

*Source*: Authors’ own work

ISWP-SWG, International Society of Wheelchair Professionals Standards Working Group; MDT, multi-drum tests; UV, ultraviolet.

The selected concept #5 (turntable system design) was drafted in SolidWorks (Dassault Systèmes SOLIDWORKS Corporation 2016) and was reviewed comprehensively in an in-person meeting with ISWP-SWG. Design recommendations (Table 3) on the turntable and caster mounting were provided by the group and were prioritised for incorporation into the design (see Figure 7).

**FIGURE 6:**
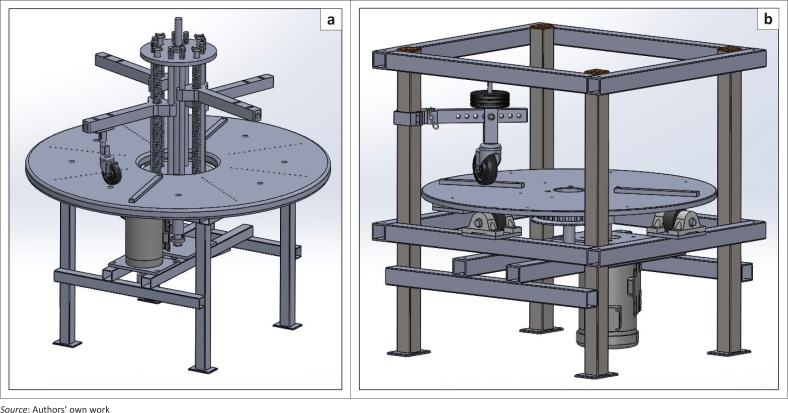
Caster assembly test design concepts #4 (left) and #5 (right).

**FIGURE 7:**
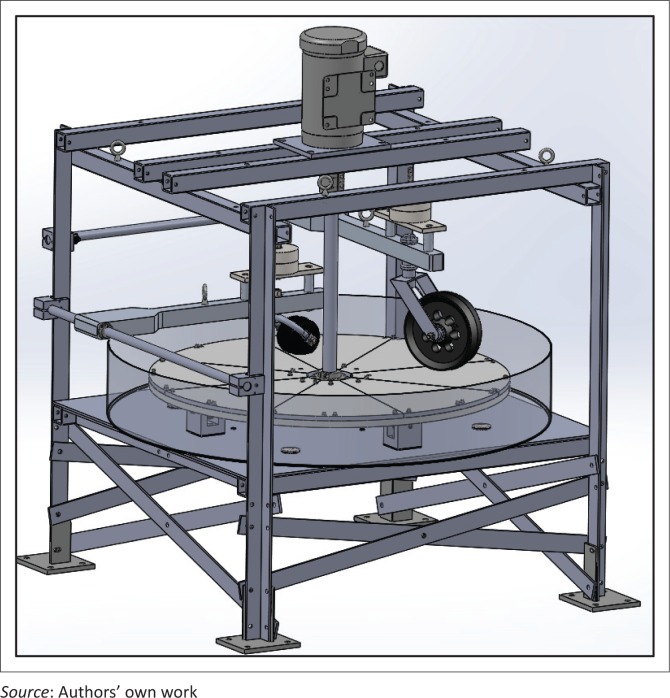
Turntable design concept.

**TABLE 3:** Design recommendations by ISWP-SWG for turntable test design.

Design features	Recommendations
1. Turntable	Larger area to accommodate four large size casters (about 8-inch diameter). Able to change the surfaces on the turntable immediately. Mount the drive motor on top of the turntable to avoid any water or dirt exposure from testing.
2. Caster arm	Weight on the caster = 30% – 35% of user weight. Variable length of suspension arm so that caster is mounted at wheelbase length of the wheelchair. Measure angle offset to the vertical and mount the caster at an angle on the arm accordingly. Clamp the rod holding the caster arm assembly on the pillars of the equipment. Use sensors to detect the descending arm following fracture of any caster assembly.
3. Design considerations for environmental test factors	Use UV lamps for aging the casters. Test the aging of rubber tires. Include gravel for testing and employ a shaker underneath. Maintain continuous agitation and level the gravel consistently. Include dirt ingress testing as per standards. Develop a tank around the table that can contain water for humidity exposure.
4. Test suggestions	Increase number of test cycles compared to multi-drum tests. Increase the height of bumps (i.e. multi-drum tests slats on drum) for testing casters. Introduce damping to eliminate caster bounce.
5. Precautions	Monitor temperature of the casters and avoid overheating them. Conduct an inspection of the casters at specific intervals.

*Source*: Authors’ own work

ISWP-SWG, International Society of Wheelchair Professionals Standards Working Group.

The authors benchmarked the design with MDT test, developed design specifications (see Table 4) and modified the design accordingly. One rotation of the turntable is twice the distance the caster would travel on MDT.

**TABLE 4:** Specifications of multi-drum tests and new caster assembly test design.

Feature	Multi-drum tests	New caster assembly test design
Speed	1 m/s	1 m/s
Test cycle	One rotation of the drum	One rotation of the turntable
Minimum number of test cycles	200 000	101 600 ≈ 100 000
Number of slat hits per revolution	1	2.02 ≈ 2
Weight on each caster	Varies between 19.5% and 35% for different wheelchairs	30% of the ISO 7176 Section 11 dummy weight = 30 lbs
Nature of caster impacts	Casters are subjected to straight/vertical impacts from slats.	Casters will be subjected to straight/angular impacts.
Wheelbase length	Varies between 15 and 23 inches for wheelchairs.	The caster arm design allows for variable positioning on the turntable. Maximum length = 28 inches. Will not accommodate wheelbase length of three wheeled chairs.
Ability to change surface	Not applicable	The turntable is equipped with eight pie-shaped pieces, which can accommodate patterns that simulate different surface types.
Number of casters tested simultaneously	2	4

*Source:* Authors’ own work

Feedback from machine shop staff at HERL was related to operation and fabrication of the test equipment. Three important suggestions were received as below.

- Include a gearbox (speed reducer) for rotating the turntable.
- Deploy corrosion resistant rollers beneath the turntable to reduce the risk of rusting.

**FIGURE 8:**
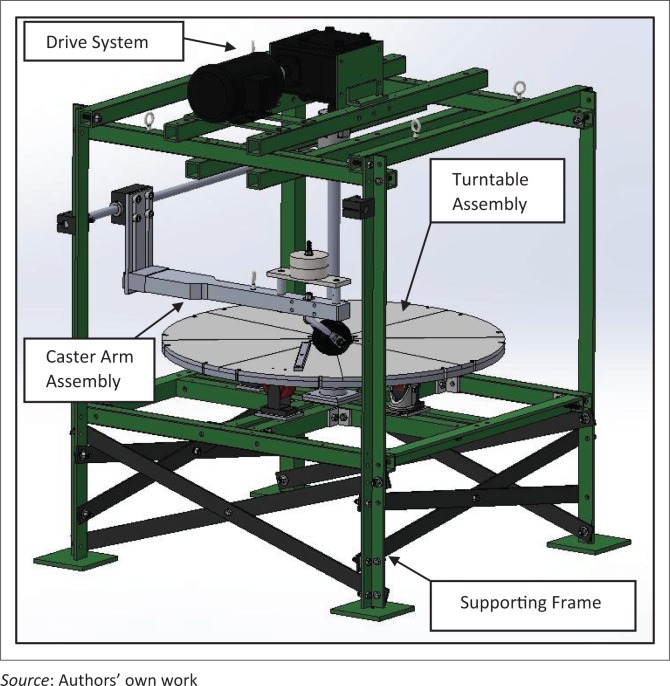
Final caster test equipment drawing for fabrication (includes only one of four possible caster support arms).

Figure 8 shows the final drawing prior to fabrication and assembly of the caster test equipment.

- Place crossbars under the turntable assembly to strengthen the equipment foundation and reduce any movement between the vertical angle iron bars.

### Caster test equipment description

The test equipment can be divided into four modular designs: (1) drive system, (2) supporting frame, (3) turntable assembly and (4) caster arm assembly.

### Drive system

**FIGURE 9:**
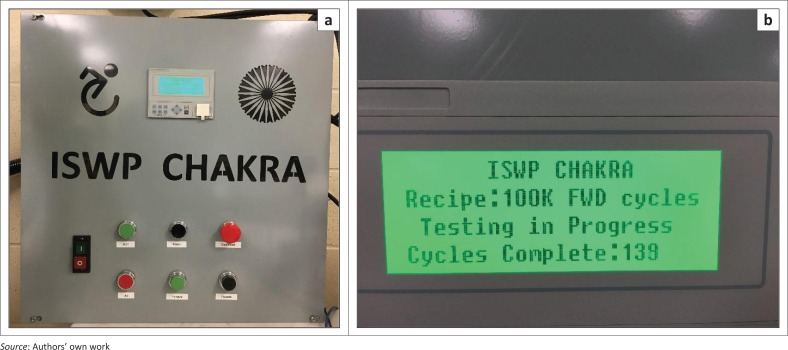
Controller box of the caster test system (left) and LCD display (right).

The drive system consists of a motor, gearbox, motor driver and system controller connected to an LCD display. The gearbox and motor selection were based on the power requirements and functionality of the test. A 2HP reversible induction motor (model# MTR-002-3BD18) from Automation Direct (Automation Direct 2016), 40:1 ratio gearbox (model# 13-325-40-R) from Surplus Center (Surplus Center 2016a), AC motor drive (model# FM50) from Teco Westinghouse (Surplus Center 2016b) and a Micro820 Programmable Logic Controller System from Allen Bradley (Rockwell Automation Inc. 2016a) were selected. The motor driver was programmed manually based on the direction and speed requirements of the turntable system, and the system controller was programmed using the Connected Components Workbench (Rockwell Automation Inc. 2016b). Three different programmes were developed – (1) one directional turntable rotation similar to MDT; (2) one directional rotation with a reverse cycle after a specific number of turns; (3) a continuous clockwise/counter clockwise rotational movement of the turntable. The LCD display shows the test programme, the status of test and the number of test cycles completed. The motor driver, the system controller and the LCD display are housed in an enclosure as shown in Figure 9.

### Supporting frame

The frame consists of four vertical angle iron bars of 4½ inch height that are connected by a web of steel square tubes and angle irons on the top and below the turntable. The top web supports the motor, gear reducer and flanged bearing, and the one below the turntable supports the rollers and shaft bearing housing. To strengthen the foundation and eliminate movement of vertical bars, flat steel crossbars have been attached.

### Turntable assembly

**FIGURE 10:**
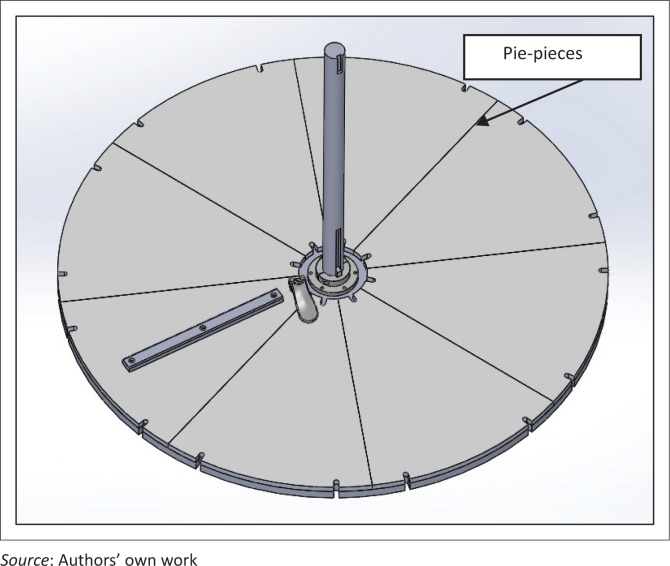
Turntable assembly with pie-pieces (only one slat mounted to pie-piece).

The turntable (Figure 10) is a 40-inch diameter circle cut from ¾ inch aluminium plate. The turntable is connected to the gearbox with a long shaft through a flange-mounted ball bearing (for support) and a Replaceable-Center Flexible Shaft coupling. The shaft is mounted on a thrust bearing under the table. Polyurethane rollers support the turntable rotation from underneath and absorb the impact from casters bouncing on the turntable. Flange couplings attach the turntable to the shaft. Pie-shaped pieces 8½ inches thick are clamped to the turntable on top. They serve as plates for accommodating different surface patterns and are currently used for holding slats at desired angles.

### Caster arm assembly

**FIGURE 11:**
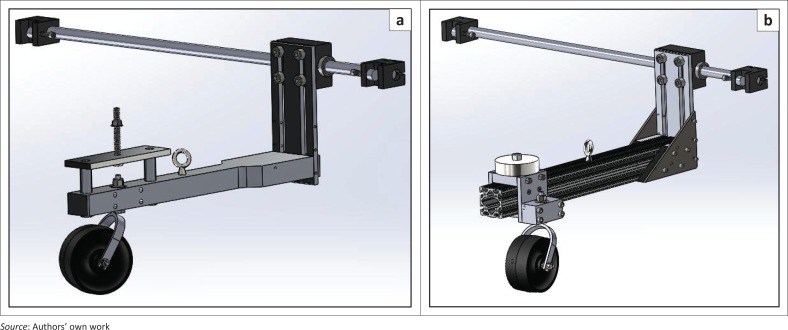
Initial design (left) and revised design (right).

Initial design of the assembly included a 2-inch thick caster arm attached to a vertical member that could slide on a block holding a steel rod as shown in Figure 11. The caster’s stem bearing assembly (to be tested) is accommodated inside a housing attached to the arm, and barbell weights are mounted on top of the arm. The arm hinges on the rod that has its ends clamped to the angle iron uprights, and the position of the rod can be adjusted vertically along the length of those uprights. The maximum wheelchair axle height that can be simulated is about 22 inches. The initial arm design was not flexible enough to position caster designs of variable diameters on the orthogonal axis of the turntable; therefore, the design was revised (see Figure 11) with 8020 components (80/20 Inc. 2016).

**FIGURE 12:**
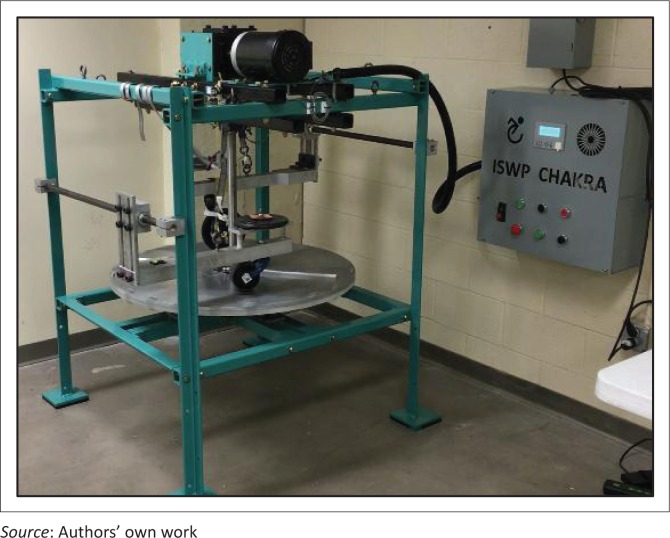
ISWP Caster Assembly Test.

Fracture failure of casters during testing causes the caster arm to collapse and fall on the turntable crushing the caster or damaging the turntable. To immediately detect the fall, a limit switch with rotating lever is mounted above each of the caster arms and strings are used to connect the lever from the limit switch to an eyebolt on the arm. For appropriate detection of failure and avoiding any damages, a safety strap is used to prevent a vertical drop of the arm after a failure and hold the arm while the limit switch is triggered. Figure 12 shows the new caster testing equipment that was fabricated and assembled. The parts were powder-coated green and black for aesthetic appeal and to reduce the risk of corrosion.

### Feasibility testing results

**FIGURE 13:**
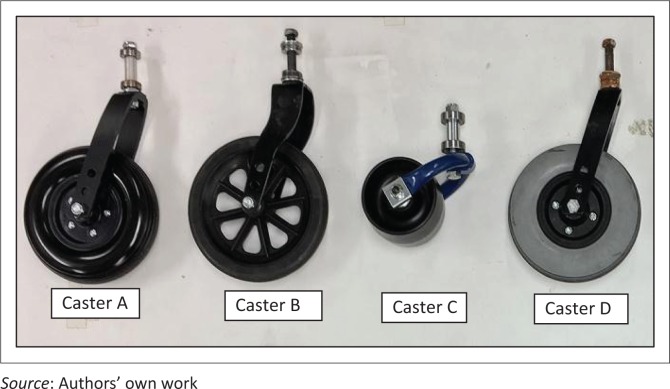
Caster assemblies tested in initial testing phase.

**FIGURE 14:**
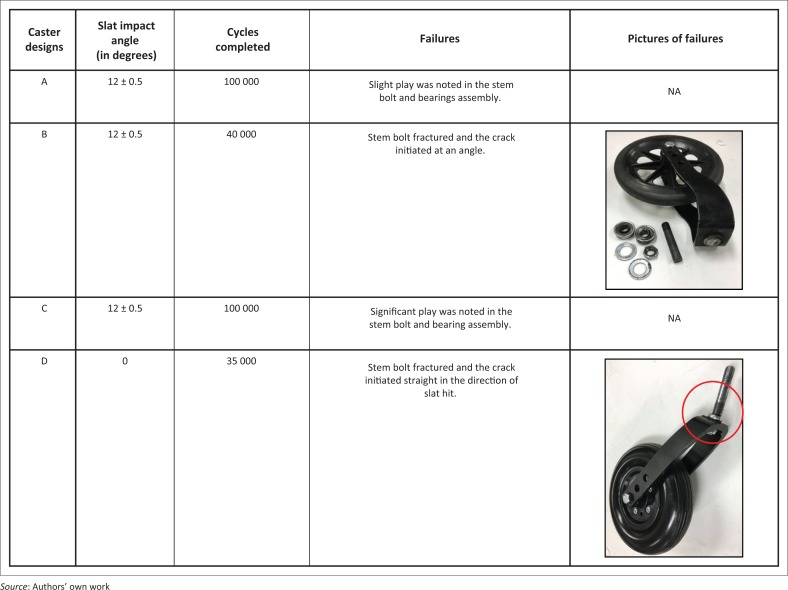
Results from initial testing of caster assemblies.

Four caster designs with solid tires as shown in Figure 13 were tested first. This initial testing to verify reliable performance of the new test equipment was conducted with 20 lbs ± 1 lbs weight on each caster and 100 000 test cycles. A total of 100 000 cycles was chosen as it is equivalent to 200 000 MDT cycles which is the minimum qualification requirement for wheelchairs (International Organization for Standardization 2014). Results from initial testing are shown in Figure 14.

### Preliminary testing results

**FIGURE 15:**
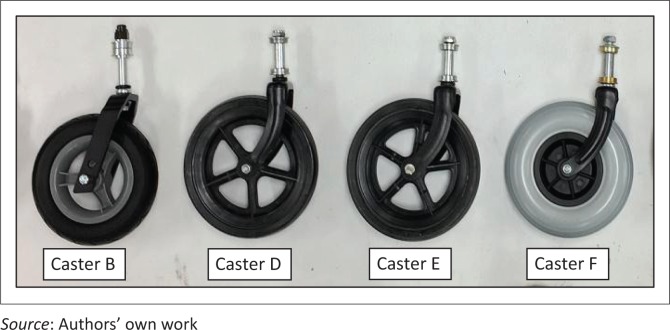
Caster assemblies tested in preliminary testing study (Model A is not shown).

**FIGURE 16:**
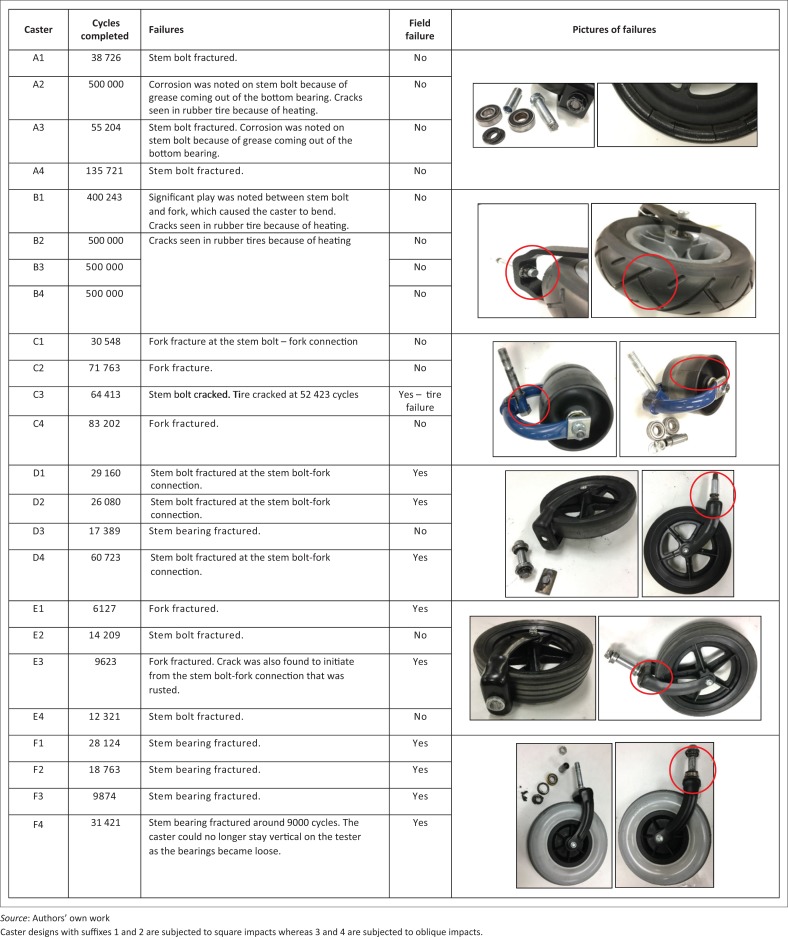
Preliminary testing results with different caster designs.

Preliminary testing to evaluate the effect of square versus oblique slat impacts was conducted with 31% ± 1% lbs weight on caster models (see Figure 15) and 500 000 cycles. Caster design C from feasibility testing was used in this study because of availability of samples. Weight selection was based on ISWP-SWG recommendations and the availability of weight plates because weight carried by casters typically ranges between 20 and 40 lbs (Caspall et al. 2013; Kauzlarich et al. 1984; Lin, Huang & Sprigle 2015; Sauret et al. 2011). A total of 500 000 test cycles was selected because MDT testing until failure is conducted until 1 million cycles, which is five times the minimum number of required cycles for testing (Cooper et al. 1996; Kwarciak et al. 2005). To avoid caster shimmy during testing, stem assemblies were tightened such that the casters would not lock and would reverse their direction smoothly when the turntable was reversed during the test setup. Additionally, for the preliminary test, the casters were placed away from the turntable centre at 11.5-inch radius which simulated about 3 years of regular travel at 1 m/s assuming an average user travels about 800 m/day (Caspall et al. 2013). Results of preliminary testing and manufacturer feedback on failures are shown in Figure 16. Among the caster models, no significant differences were found between the number of cycles completed with square and oblique slat impacts (*p* > 0.05).

## Discussion

Less-resourced environments demand greater durability from wheelchair products. For this reason, the WHO Guidelines recommend rigorous quality evaluation through tests that simulate LRE conditions (Borg & Khasnabis 2008). Development of such tests has been undertaken by the ISWP-SWG and the group has prioritised caster durability testing owing to frequent caster failures.

Review of testing methods for casters showed that several standard tests are available. ISO 7176 durability tests – MDT and CDT – are tests with reportedly good repeatability and subject casters to square slat impacts. Drum tests developed by wheelchair manufacturers for testing casters separately are similar. ISO and ICWM caster testing standards have been published for institutional and industrial casters but they are not applicable to outdoor use of casters with wheelchairs. Testing methods reported in literature test casters for durability with mechanical loads and impacts only. These methods did not include exposure to environmental and use conditions as seen in LREs which also contribute to the degradation in product quality and consequently failures. None of these tests simulated exposure to different surfaces, high temperature, humidity or ultraviolet (UV) light. Deploying such test factors in lab-based accelerated tests is important to reproduce accurate product lifecycle and failures.

Evaluating caster durability using a new testing method with relevant testing conditions was proposed by the ISWP-SWG. Based on previous experiences, experts deemed it difficult to revise the current ISO testing setup. Issues noted with such modifications were related to securing different surface patterns on the cylindrical surfaces of MDT drums and enclosing the testing setup in a chamber for environmental testing. Thus, the development of a new caster testing system was initiated. Functional requirements specified for the new system were largely based on the need for inclusion of environmental testing factors that were lacking in existing testing methods. Design concepts that were variations of the reviewed testing methods (ICWM standards) with additional design features were considered.

Of the design concepts proposed for selection, the turntable test design addresses several functional requirements and offers several advantages. Implementing testing factors relevant to LRE conditions seems feasible on the setup especially with exposure to different surfaces. Pie-shaped pieces have the capability to incorporate various surfaces patterns that correspond to uneven terrains and are representative of exposure to muddy ground, gravel, sand and dirt. With the availability of eight pieces, casters can be exposed simultaneously to different surface types. These pieces are clamped to the turntable such that they can be replaced easily in minimal time if change in surface exposure is required. Slats can be attached at different angles for simulating straight and oblique hits from bumps and obstacles. Exposure to different surfaces and loads conditions in LRE is responsible for failures related to tire wear and cracking, and inducing low-cycle fatigue effects in caster parts such as the stem bolt, wheel and bearings. To reproduce such failures, new surface patterns and testing protocols, which are validated to actual outdoor exposure, need to be developed for the new turntable testing setup.

Caster stem assembly failures are common during field use and standard testing. The quality of stem bearings can be evaluated on the new caster testing equipment because the turntable can reverse its direction – unlike MDT, which has casters run in the direction of wheelchair primary propulsion. The stem bearings can be tested by either having the casters roll in the opposite direction for a certain number of cycles after designated number of forward cycles or having casters continuously change direction with to and fro rotating motion of the turntable.

The new test design allows caster exposure to environmental testing factors such as moisture exposure and UV light. To incorporate moisture exposure, immersing casters partially in water in a tank surrounding the turntable was suggested by the ISWP-SWG members. The water tank addition to the design is yet to be implemented because of concerns regarding controlling for water characteristics such as temperature, oxygen content, level of exposure (which may not be repeatable) and risk of corroding of testing equipment. Another suggestion by one of the authors was deploying water sprinklers, which can draw water from a tank under controlled conditions and assist in reducing caster tire temperature during testing. Corrosion of caster assembly parts is a top concern, and the failure can be simulated on the turntable design with reasonable modifications. For simulating rapid aging, UV/heat lamps can be mounted beside each caster in an enclosure. Degradation of rubber tires and other plastic materials can be simulated in this manner.

In sum, the caster testing equipment developed by ISWP-SWG caters to most functional requirements and has the capability to simulate different testing factors. To incorporate these factors, the test setup requires upgrades as stated previously.

Four different caster designs were tested to evaluate the feasibility of testing casters with the new system. Two caster models (one from a LRE wheelchair) completed the minimum number of test cycles, and the others experienced stem bolt fractures with crack initiation at an angle because of oblique hits. These fractures were anticipated failures as they are often seen with MDT. Reliable testing with the initial set of casters motivated testing caster assemblies for evaluating the effect of straight versus oblique impacts. The preliminary testing with models from LRE wheelchairs (except model B) revealed that slat impact angles do not have any effect on caster durability. However, it should be conceded that the sample size for testing against each condition was small, which could have led to a non-significant result. Also, oblique impacts on the caster can have a lesser effect compared with square impacts, as the stem bearings allow the casters to swivel moderately to accommodate the impact. While there was no significant difference in number of cycles between two conditions for each model, the types of failures were relatively consistent.

Caster size was one of the differentiating factors among models during preliminary testing. Casters A and C have smaller diameters comparatively, and model A samples were found to be significantly affected from slat impacts causing stem bolt fractures. Stem bolt fractures are typical in caster assembly failures with MDT testing as noted in several wheelchair testing studies (Cooper, Stewart & VanSickle 1994, Cooper et al. 1996, 1997, 1999; Fitzgerald et al. 2001). These fractures initiate from the bolt surface where the bolt connects with the fork because there is only a minimal cross-sectional area to withstand the moment-force from impacts. Another cause for such failure can be grease leakage from lower stem bearings (seen with two casters) that rusts the lower part of the stem bolt and inner bearing rings. The rust-affected area can initiate cracks from the surface. The manufacturer of the model A caster disagreed with the test results as stem bolt failures have not been seen during field use.

The strength of caster models was affected by design factors such as tire thickness and hardness as well. While model A tires are pliable, the casters suffered greatly from slat impacts because the sidewall tires are not as thick. All large size casters had significantly greater sidewall thickness, and among them model B was found to be more durable because the tires were able to absorb impacts. Tire hardness for caster B and F is 70A while other models have a hardness ranging from 78A to 90A. Hard tires were found to transmit the moment-forces from impacts directly to the stem bolt and fork connection, which causes bolts to shear. Model B caster parts (especially tire and fork) were reportedly expensive and high-quality and, hence, the casters endured the slat impacts without significant failures. However, this model did experience tire cracking failure after 500 000 testing cycles because of excessive heating of rubber. Tire failures such as cracking and wearing are common in the field with model C casters as per the manufacturer. The caster test was able to simulate this failure for model C in the laboratory; however, it should be noted that there are several other outdoor conditions apart from mechanical stress and heat that cause this type of failure.

A few caster models were found to undergo fork fractures during testing. Three caster C models, despite their sturdy rubber tire design for absorbing impacts, suffered fork fractures. On this model, part of the fork that accommodates the stem bolt includes a thin tube piece and a bent flat metal piece welded to the rest of the fork. This welded assembly is constantly under tension. Thus, the fork design is found to shear from fatigue caused by slat impacts. The manufacturer of caster C reported that they have not witnessed fork fractures in the field. Model E incurred two fork fractures. Its polyurethane tires have 90A hardness and suffer significantly from impacts. The cross-section of the fork where the prongs connect with the centre piece of the fork has less material thickness, which is a cause for fracture. Models D and E are from the same manufacturer and have different tire designs and materials. The design of the stem bolt and fork connection is the same; the stem bolt is welded to a metal piece that holds the bolt against the fork. This welded connection was found to be a pain point because the weld cannot endure fatigue. The connection breaks prematurely, initiating a bolt or fork fracture. The rubber tires (85A hardness) on model D experienced rubber chalking which may cause them to gradually thin and eventually etch or crack. The manufacturer of models D and E acknowledged that the failures from preliminary testing were witnessed in the field occasionally, but they were not observed during MDT and CDT. Retesting the casters after an upgrade to the fork design was suggested to the manufacturer.

Stem bearing fractures were observed during preliminary testing, particularly with model F. The top stem bearing is a flanged cup thrust bearing that should accommodate vertical thrust from the caster. However, the outer ring material is made of low-strength steel, which causes these bearings to rupture. This failure happened quite early during testing and the casters were taken off only after they could no longer run vertically straight. The manufacturer of caster F admitted that bearing fractures have been noted in the field but they have happened after a year or two of use.

Overall, the failures observed with 50% caster models during the preliminary testing study were representative of their field failures. Some manufacturers mentioned that their models mostly undergo wear failures in the field, rather than fracture failures. These wear failures can be attributed to rough terrain and environmental factors that wear down the tires and bearings specifically. To reproduce wear failures, the inclusion of additional test factors in the testing protocol is necessary. Further, the study results also led the manufacturers to comment that the caster test is more rigorous than standard tests as a majority of the casters failed before 100 000 test cycles which is nearly equivalent to 200 000 MDT cycles (representing 3–5 years of outdoor use). The high magnitude shocks on the caster test result from slat impacts are nearer to the centre of the caster compared to MDT; however, these shocks assisted in exposing the weak links in the caster during the study. Failures such as bearing and fork fractures that were witnessed in the field were missed by manufacturers during their standards testing. The high magnitude shocks may be characteristic of LRE outdoor use which led to representative fracture failures. Still, the feedback about the rigorous nature of the test mandates validation of the test to outdoor shock exposure that can assist in predicting fracture failures more accurately. The ISWP-SWG plans to conduct a series of validation experiments which will be followed with upgrades to the test equipment and testing protocol.

### Limitations

The caster assembly testing was prioritised and developed as a part of additional wheelchair tests based on consensus from the ISWP-SWG members, which may cause potential expert bias in this study. The caster assembly testing equipment has been developed for testing against several quality-affecting factors; however, casters in the study were only subjected to load testing. There are certain design shortcomings with the equipment:

- The three-wheeler casters have a longer wheelbase, which cannot be simulated because of space restrictions.
- Exposing the caster to quality-degrading factors like corrosion, high temperatures and UV may potentially cause the testing equipment parts to degrade faster.
- Currently, the casters experience a slight bounce after slat impacts as compared to outdoor use. Deploying shock absorbers on the arms can mitigate this.

### Future work

The caster test performs durability testing of casters consistently and requires further upgrades for incorporating additional testing factors. The authors plan on validating the shock exposure on caster testing to outdoor shocks by analysing forces and corresponding fatigue experienced by casters in the field, and developing different surfaces on the pie-shaped pieces to replicate the effect. This validation will be followed by the addition of more test factors. For integrating corrosion and environmental wear, casters will be subjected to a cascading testing approach. For example, casters can be exposed to humid conditions for corrosion effect followed by UV exposure at an elevated temperature and then conducting accelerated durability testing with slats or other surfaces. It is anticipated that the integration of additional test factors will produce failures that are representative of field failures. The ISWP-SWG plans to integrate the caster test into ISO standards as a new or add-on standard to ISO 7176 or as a technical specification so that they are harmonised with national standards.

## Conclusion

Wheelchair casters experience premature failures in the LREs based on research and anecdotal evidence, which motivated development of a new caster durability test. Simulating the demanding outdoor conditions requires the employment of several testing factors during durability testing which are not included in any existing caster testing standards. We reported the results of a newly developed test for evaluating caster assemblies, which have the capability to expose casters to different testing conditions that are necessary for additional testing of wheelchairs as recommended by WHO Guidelines. The test conducts durability testing similar to MDT currently, but on the caster system alone (rather than the whole chairs) and offers a range of additional testing conditions, such as debris, rough terrain and moisture. Future work includes implementing these additional testing conditions and comparing failures on the caster test to field failures.
